# Docetaxel-Induced Pneumonitis in a Patient With Metastatic Lung Adenocarcinoma

**DOI:** 10.7759/cureus.67939

**Published:** 2024-08-27

**Authors:** Brian Chua, Yi Hern Tan

**Affiliations:** 1 Respiratory & Critical Care Medicine, Singapore General Hospital, Singapore, SGP

**Keywords:** drug-induced pneumonitis, oncology, diffuse lung disease, drug-induced interstitial lung disease (di-ild), docetaxel

## Abstract

Docetaxel is a taxane anti-neoplastic agent commonly used in the treatment of solid-organ tumours. Here, we describe a case of a patient with metastatic lung adenocarcinoma who had disease progression following initial treatment with a combination of pembrolizumab, pemetrexed and carboplatin. She received three cycles of docetaxel and had a favourable oncological response but was admitted for breathlessness following the third cycle. A repeat computed tomography scan of the thorax showed predominantly right-sided ground-glass opacities and consolidation. The patient underwent high-risk bronchoscopy and bronchoalveolar lavage. Once infection was confidently ruled out, she was started on high-dose steroid therapy and responded to treatment.

## Introduction

Drug-induced pneumonitis can present a significant diagnostic challenge because symptoms, radiographic findings and even findings from BAL fluid can be non-specific. The diagnosis of drug-induced pneumonitis requires rigorous interrogation of drug history and exclusion of other causes. Taxanes are commonly used in the treatment of solid-organ tumours [1]. Here, we present a case of docetaxel-induced pneumonitis in a patient who required mechanical ventilation and responded to high-dose methylprednisolone.

## Case presentation

A 60-year-old woman was diagnosed with metastatic lung adenocarcinoma in December 2020. Molecular profiling showed TP53 mutant, MET polysomy, PD-L1 TPS 15%. The initial sites of disease included thoracic and abdominal lymph nodes, pleura, bone and bilateral upper lobes. She was started on carboplatin, pemetrexed and pembrolizumab but unfortunately, her disease progressed after five cycles of therapy. She was switched to IV docetaxel 75mg/m^2^.

**Figure 1:**
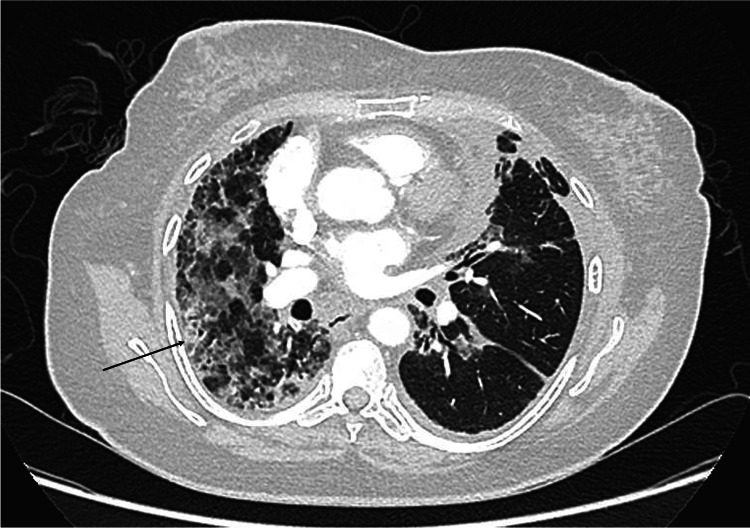
Computed tomography (CT) scan of the thorax done after three cycles of docetaxel, showing patchy areas of ground-glass and consolidation, predominantly in the right lung (indicated by the black arrow)

After three cycles of docetaxel, a repeat CT thorax, abdomen and pelvis was performed for re-staging purposes which showed reduction of the disease burden. The CT scan was however significant for new areas of consolidation and ground-glass opacities in predominantly the right lung, with much lesser involvement of the left lung (Figure 1).

**Figure 2:**
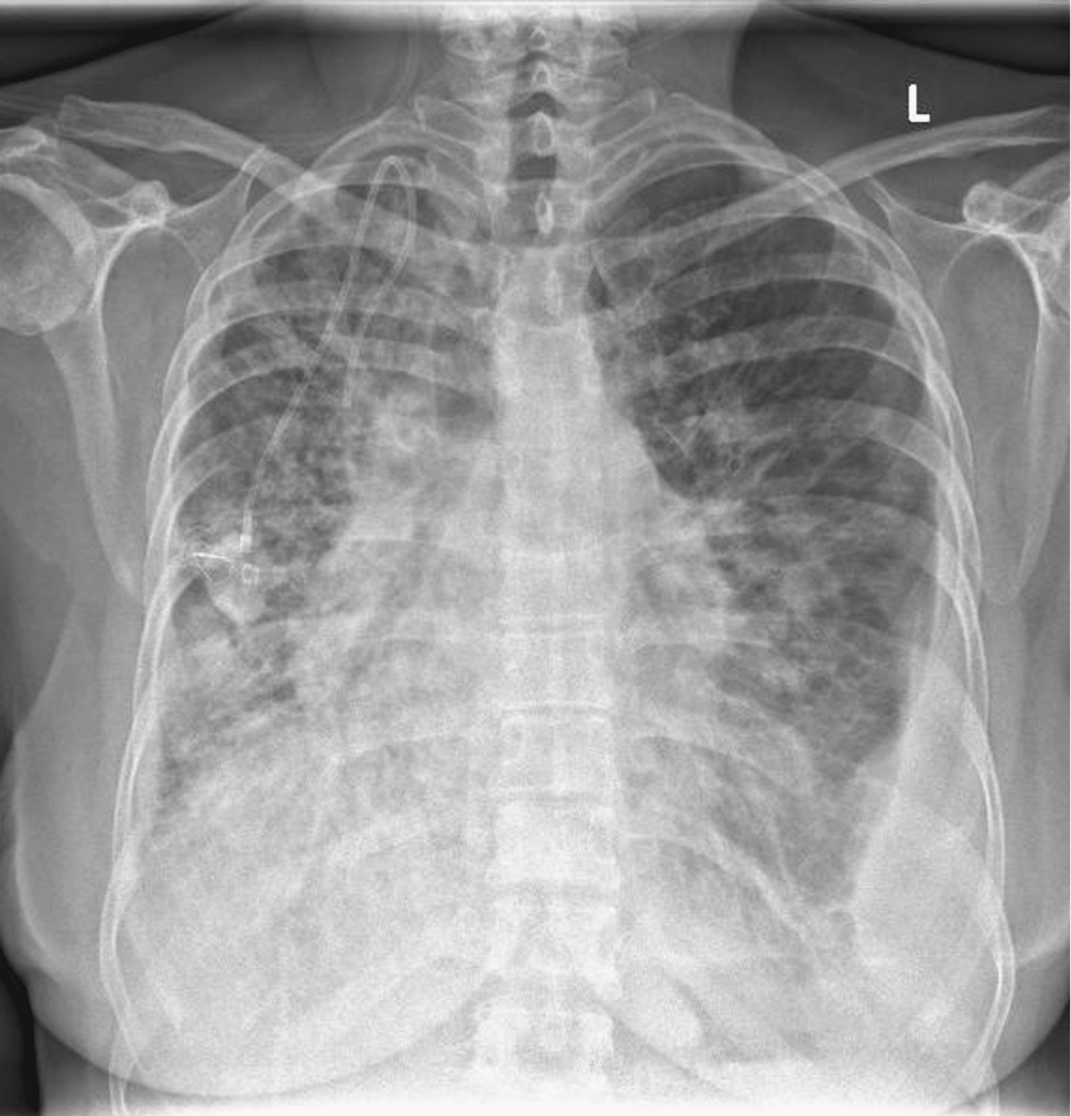
Chest radiograph showing interval development of bilateral hazy infiltrates over both lung fields

She was advised to return to the Oncology clinic for evaluation but before her scheduled appointment, she presented to Emergency Department with worsening dyspnea. Upon admission, she was febrile and haemodynamically stable. Her oxygen saturation was 95% on intranasal oxygen at 2L/min. There were bilateral coarse crepitations on auscultation with no peripheral oedema. Inflammatory markers were raised: procalcitonin 0.07ug/L and C-reactive protein 35.7mg/L. The total white blood cell count was 9.59 x 109/L. The chest radiograph showed interval development of bilateral hazy infiltrates (Figure 2).

She was referred to Respiratory Medicine and the differential diagnosis included infective causes and docetaxel-induced pneumonitis. She was empirically covered with IV piperacillin-tazobactam and was started on IV hydrocortisone 100mg Q6H. She did not improve following three days of empiric therapy with antibiotics and anti-inflammatory agents and remained oxygen dependent. She was counselled for and underwent a high-risk bronchoscopy and bronchoalveolar lavage (BAL). Forty-eight hours after the bronchoscopy, she continued to deteriorate and required invasive mechanical ventilation (IMV) in the intensive care unit (ICU).

On day 2 of her ICU admission, her preliminary microbiological cultures, including Pneumocystis Jirovecii microscopy and polymerase chain reaction (PCR) test, acid-fast bacilli smear, tuberculosis PCR, aerobic culture, fungal microscopy and culture, Galactomannan test and cytomegalovirus antigen from her BAL, were negative. She was started on pulsed IV methylprednisolone 500mg once a day for three days. Antibiotics were empirically escalated to IV meropenem to cover for pyogenic causes of severe pneumonia. She was ventilated with lung protective strategies including, low tidal volumes (4-6ml/kg), permissive hypercapnia. Ventilator requirements improved, the patient passed a spontaneous breathing trial and was extubated after six days of IMV.

**Figure 3:**
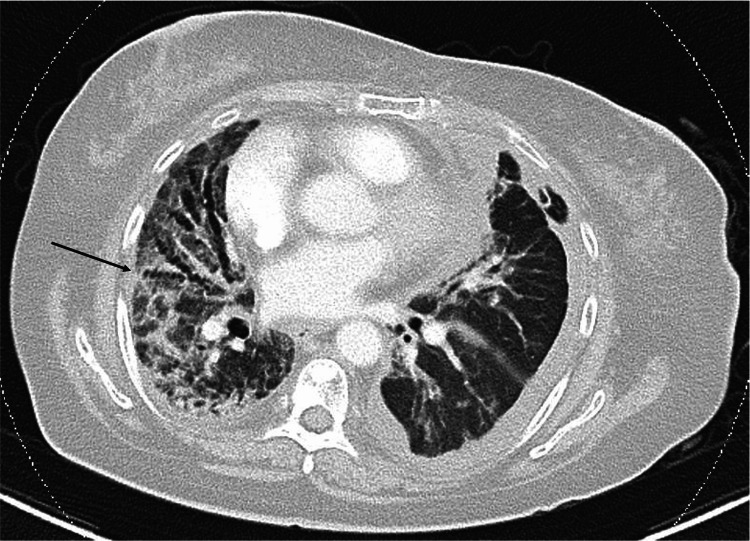
Computed tomography (CT) scan of the thorax done on D60 of admission, showing interval improvement of bilateral consolidation, with residual fibrosis (traction bronchiectasis and reticular infiltrates, as indicated by the black arrow)

After completing three days of IV methylprednisolone, she was started on prednisolone 60mg once a day and the dose was tapered by 10mg every week. Subsequently, she underwent inpatient rehabilitation and was completely weaned off supplemental oxygen before discharge from the hospital. A repeat CT scan of the thorax showed interval improvement of her bilateral consolidative changes with residual areas of lung fibrosis (Figure 3).

In view of poor response to first-line anti-neoplastic therapy, and significant toxicities from docetaxel, no further oncological treatment was advised. The patient remained clinically stable for two months but was unfortunately readmitted for severe pneumonia and passed away shortly after.

## Discussion

Docetaxel is a taxane anti-neoplastic agent, commonly used to treat solid-organ tumours, including non-small cell lung cancer. There have been case reports of docetaxel-induced pneumonitis and it is reported that 1-5% of patients who receive docetaxel are at risk of developing pneumonitis of grade 3 or higher [1]. Taxanes cause proliferation of cytotoxic T-cells, which can result in hypersensitivity pneumonitis of the lungs [2]. However, the exact mechanism of pulmonary injury remains unclear.

Pneumonitis has been described to occur after a few cycles of treatment, usually after the second cycle [3]. However, they can also present acutely after initiation of treatment. Common symptoms include dyspnea and cough. CT is the imaging modality of choice in patients with suspected drug-induced pneumonitis [4]. However, radiological abnormalities can be non-specific. Drug-induced pneumonitis most commonly presents as bilateral peripheral ground-glass opacities, with/without consolidation, with basal predominance [4].

The role of bronchoscopy remains equivocal in the diagnosis of drug-induced pneumonitis. The primary objective of BAL is to rule out alternative processes, including infection, alveolar haemorrhage or metastatic dissemination of the underlying cancer. Cell counts might reveal a lymphocytic, eosinophilic pattern but this is not specific to drug-induced pneumonitis [4-6]. Lung biopsies are often reserved for patients with progressive lung disease despite cessation of taxane therapy when the cause of pneumonitis is uncertain.

Management strategies include discontinuation of the suspected offending drug(s) and administration of glucocorticosteroids. There is no consensus on the dosing of corticosteroids in this setting. Steroid regimes published in the literature include prednisolone started at doses of 0.5 to 1mg/kg/day and tapered according to the clinical response or IV methylprednisolone in severe cases [7]. Patients with peripheral eosinophilia might respond better to steroids [2]. Limited data suggests that the mortality for taxane-induced pneumonitis may range from 40% [3] to 80% in patients requiring mechanical ventilation [2].

## Conclusions

Drug-induced pneumonitis is a rare adverse reaction of docetaxel. Physicians should be mindful of this complication, especially if patients fail to respond to empirical antimicrobial therapy. Early diagnosis is key as mortality is high in patients with advanced disease. There may be a role for bronchoscopy to rule out alternative diagnoses such as infections, alveolar haemorrhage or metastatic disease. Treatment of docetaxel-induced pneumonitis requires suspicion of the offending agent and early initiation of corticosteroids. 
